# Is the Erector Spinae Plane Block Effective for More than Perioperative Pain? A Retrospective Analysis

**DOI:** 10.3390/jcm11164902

**Published:** 2022-08-21

**Authors:** Uri Hochberg, Silviu Brill, Dror Ofir, Khalil Salame, Zvi Lidar, Gilad Regev, Morsi Khashan

**Affiliations:** 1 Division of Anesthesiology, Institute of Pain Medicine, Tel Aviv Sourasky Medical Center, Tel Aviv 6423906, Israel; 2 Sackler School of Medicine, Tel Aviv University, Tel Aviv 6997801, Israel; 3 Spine Surgery Unit, Neurosurgical Department, Tel Aviv Medical Center, Tel Aviv 6423906, Israel

**Keywords:** chronic pain, cancer-related pain, ESP block, pain management, ultrasound-guided

## Abstract

**Introduction:** The thoracic Erector Spinae Plane Block (ESPB) is an ultrasound-guided block that has gained popularity and is widely used in acute pain setups. However, data regarding its role in chronic and cancer-related pain are anecdotal. **Material and Methods:** The study is a retrospective analysis of patients who underwent ESPB. The cohort was divided into subgroups based on three determinants: etiology, pain type, and chronicity. **Results:** One hundred and ten patients were included, and genders were affected equally. The average age was 61.2 ± 16.1 years. The whole group had a statistically significant reduction in a numerical rating scale (NRS) (7.4 ± 1.4 vs. 5.0 ± 2.6, *p*-value > 0.001). NRS reduction for 45 patients (41%) exceeded 50% of the pre-procedural NRS. The mean follow-up was 7.9 ± 4.6 weeks. Baseline and post-procedure NRS were comparable between all subgroups. The post-procedural NRS was significantly lower than the pre-procedural score within each group. The proportion of patients with over 50% improvement in NRS was lower for those with symptom duration above 12 months (*p*-value = 0.02). **Conclusions:** Thoracic ESPB is a simple and safe technique. The results support the possible role of ESPB for chronic as well as cancer-related pain.

## 1. Introduction

Chronic pain is a common, complex, and distressing condition that impacts many aspects of patients’ health and quality of life. Therefore, it can place a significant burden on patients and on the broader healthcare system. The Global Burden of Disease Study of 2016 reaffirmed that the high prominence of pain and pain-related diseases is the leading cause of disability and disease burden globally [1,2].

The Erector Spinae Plane Block (ESPB) belongs to a growing number of ultrasound-guided blocks that aim to deliver an analgesic solution to the soft tissue, or the interfascial plane, as opposed to the classic method of a direct nerve block. Shortly after its first description, the ESPB was adopted in clinical practice for multiple types of thoracic, abdominal, and extremity surgeries [3]. It is regarded as an effective, safe, and simple method for acute pain management [4]; however, despite its popularity, both the mechanism of the block and the extent of injectate spread are unclear [5].

Thoracic origin chronic pain represents a particular challenge as the interventional aspect requires its own special consideration. As with all neuraxial techniques, thoracic neuraxial procedures have contraindications and limitations (for example, coagulopathies, thoracic spine deformations, patient refusal) and other possible complications with considerable potential morbidity, including pneumothorax and neurovascular damage.

Although the first description of the block was for chronic neuropathic pain, most publications are concerned with acute pain management. The current literature addressing its use for chronic and cancer-related pain is scarce and mostly anecdotal in nature [6,7,8,9,10].

In this paper, we described the outcomes of the ESPB applied to patients at our pain institute, a regional referral center within a tertiary, university-affiliated medical center. We compared outcomes between different groups of patients based on the origin of the pain, type of pain, and symptom duration.

## 2. Materials and Methods

### 2.1. Study Design

A retrospective analysis.

### 2.2. Setting and Study Population

The study was conducted at the Pain Institute Center of the Tel Aviv Souraski Medical Center and was approved by the hospital’s ethics committee (No. 0003-20-TLV). A retrospective review was carried out on the medical records of all patients who underwent thoracic ESPB between October 2018 and August 2021.

All participants provided written informed consent prior to undergoing the procedure, similar to other invasive procedures in our center. Inclusion criteria included: age of 18 years or above, diagnosis of thoracic back pain, a numerical rating scale (NRS) for pain ≥ 6, no significant motor weakness, no signs or symptoms of myelopathy, failure of other conservative treatment (i.e., physical therapy and oral analgesics), and at least a one-month post-procedure follow up.

Exclusion criteria: Allergy or hypersensitivity to steroid or amide local anesthetics, pregnancy, and breastfeeding.

### 2.3. Thoracic ESP Block Technique

The procedure was carried out under ultrasound guidance. The patient was placed in a prone position, and the ultrasound probe was set 2.5–3 cm laterally to the spinous process at the desired thoracic vertebral level on a parasagittal plane. Normally, a high-frequency linear probe was used. In the case of obese patients, a curvilinear (2–5 MHz) probe was used. Under ultrasound guidance, a 22 G needle measuring 50 or 100 mm was then inserted in a craniocaudal direction using the in-plane technique. The injection takes place at the fascial plane, deeper into the erector spinae muscle group. The solution injected was 10 mL of 1% lidocaine with dexamethasone 10 mg for a unilateral injection and 15–20 mL of 1% lidocaine with dexamethasone 10 mg for a bilateral injection.

### 2.4. Data Collection

Pre-procedural baseline demographic, clinical, and imaging data were collected. Demographic and procedural variables included: age, gender, pre-procedural NRS, and duration of symptoms at the first visit. Procedural variables included the side and level of the injection. Post-procedural variables included post-procedural NRS scores and the total number of ESPBs performed. Adverse effects were also monitored and documented. The data were collected and recorded by the pain physician in charge of the patient at the pain institute.

Thoracic pain was defined as pain experienced in the thoracic area, between the T1 and T12 boundaries, and across the posterior aspect of the trunk [11].

The main outcome measured was the change in pain intensity which was assessed using an 11-point numerical rating scale (NRS), with a range from 0 (no pain) to 10 (worst possible pain). In order to evaluate clinical significance, we used a minimal clinically important change (MCID) of 2.5 [12] for NRS, and we looked at a patient with more than 50% reduction in baseline NRS.

We divided the patients into different groups based on etiology, dominant pain type, and pain chronicity. For etiology, we divided the patients into two groups: one with patients suffering from cancer-related pain and the other with patients suffering from non-cancer-related pain. Cancer-related pain was defined as pain originating directly from a thoracic neoplastic lesion.

Regarding the dominant pain type, we divided the patients again into three groups according to the pain type: nociceptive, neuropathic, and mixed pain. The neuropathic pain type was defined using strict criteria, following the current International Association for the Study of Pain (IASP) definition of “definite neuropathic pain” [13]. In cases where a discrete pathophysiological classification of pain was not either purely neuropathic or purely nociceptive, a “mixed type” diagnosis was given [14].

In order to analyze the effect of chronicity, the cohort was divided into three groups according to the duration of symptoms: up to four months, four to twelve months, and more than twelve months.

The patient clinical assessments were conducted before the procedure and at the post-procedure follow-up appointments. The pre- and post-procedural NRS were compared in the entire group and within each of the sub-groups.

At the post-procedure follow-up, patients with motor neurological deficits were referred for further surgical evaluation. Patients with significant pain (NRS > 6) were offered a second thoracic ESPB. A maximum of three procedures were allowed for each patient. Patients with no significant pain (NRS < 4) at the follow-up visit, were either discharged or offered an additional second follow-up.

### 2.5. Statistical Analysis

The statistical analysis was performed using SPSS version 19 (IBM Corp., Armonk, NY, USA). Significant differences between the groups were determined using one sample *t*-test, the X^2^ test, and the Fisher exact test to evaluate categorical variables’ independence. ANOVA and independent *t*-test were used to compare NRS values between the symptom duration groups. Pre-procedure to post-Procedure NRS changes within each group were analyzed with paired *t*-tests. A *p*-value < 0.05 was considered statistically significant.

## 3. Results

### 3.1. Participant Characteristics

One hundred and ten patients underwent the procedure, and both genders were affected equally. The average age was 61.2 ± 16.1. Sixty-one (55%) patients underwent unilateral injections, and 49 (45%) underwent bilateral injections (Table 1). Seventy-two patients (65%) were discharged from the pain clinic after one injection, and 35 (32%) patients were discharged after two injections. The most common level of injection was T3 (26 patients) (Table 2).

### 3.2. Demographic and Preprocedural Variables

When observing the etiology groups, no significant difference was found between patients with cancer-related pain and patients with non-cancer pain (Table 1). As for the pain type groups, we found significant differences in the side of injection (Table 1). In the mixed pain group, no patients received bilateral injections (*p*-value = 0.003). Analysis of pain duration groups showed a significant difference in the distribution of the injection site (*p*-value < 0.001) (Table 1).

### 3.3. Average Pain Intensity

A statistically significant reduction in NRS was found when the mean pre- and post-procedural NRS were compared across the entire cohort (*p*-value > 0.001). In fifty-eight (53%) patients, the NRS improvement exceeded the MCID, and in 45 (41%), it exceeded 50% of the pre-procedural NRS (Table 3).

The etiology group comparison showed comparable pre-procedural NRS. The post-procedural NRS was significantly lower than the pre-procedural score within each etiological group. The proportion of patients who achieved improvement higher than MCID as well as above 50% of the baseline NRS was higher in the non-cancer related group, yet, without statistical significance (*p*-value = 0.51) (Table 3).

Comparing the pain type groups showed comparable pre- and post-NRS scores. However, in the mixed pain group, the improvement in NRS did not reach statistical significance (*p*-value = 0.334) (Table 3).

In the pain chronicity groups, the pre- and post-NRS were comparable between the groups, and the improvement in these scores was found to be statistically significant within each group. The proportion of patients with more than 50% improvement in NRS was significantly lower in patients with symptoms duration of more than 12 months (*p*-value = 0.02) (Table 3).

There were no major adverse effects reported. The main adverse effect was injection site soreness. Other adverse effects reported were systemic response attributed to steroid exposure, none requiring hospitalization.

## 4. Discussion

Thoracic spine pain is prevalent, affecting about 20% of people in their lifetime. However, research related to thoracic pain is sparse compared to lumbar and cervical spine pain [11].

Thoracic ESPB has gained popularity since its introduction and is being widely used in acute pain setups; however, data regarding its role in chronic pain are mostly anecdotal. 

In this work, our purpose was to evaluate the role of ESPB in the management of chronic and cancer-related thoracic pain. We compared the outcomes based on three fundamental determinants: etiology, the dominant pain quality, and the chronicity of pain.

Our results are consistent with those of previously published reports describing the possible benefits of ESPB [6,7,8,9,10]. The mean reduction in NRS in our study was 2.4 points (*p*-value > 0.001), with 53% reporting NRS improved by more than 2.5 points, and 41% with NRS score improved by more than 50% compared to pre-procedure score. In our study, a successful, clinically meaningful procedure was defined as either a reduction in the NRS score by 2.5 or more points [15] or as a reduction of 50% compared to baseline NRS. This strict threshold, which was also selected by other trials [16,17], was chosen over the more common 30% improvement in NRS score to exclude the potential placebo effect.

Cancer patients frequently suffer from a wide range of other symptoms, and the multi-factorial causes result in a ‘‘total pain experience” [18]. As such, an etiology-based sub-group analysis was carried out, comparing patients with cancer-related pain to patients suffering from pain caused by other conditions. The first sub-group included all patients with an active neoplastic disease that causes intractable pain that is refractory to a medical regimen treatment. The proportion of patients who achieved improvement exceeding the 50% of the baseline NRS was higher in the non-oncological patients, showing a strong tendency toward statistical significance (*p*-value = 0.51).

Of the 66 cases of non-cancer-related pain, 33 were nociceptive (50%), 28 were neuropathic (42.4%), and 5 (7.6%) were mixed pain types. The nociceptive group included pain resulting from vertebral or rib fractures, deformations including kyphosis and scoliosis, soft tissue myofascial pain [19], degenerative changes in the disc and the facet joints, and bones lesions including hemangiomas. Within the 33 cases of the nociceptive group, 11 cases were due to degenerative spinal changes, 7 were myofascial pain, 6 cases were due to pain secondary to osteoporotic thoracic vertebrae fracture, 4 cases were due to consistent pain after the fracturing of ribs, 3 cases were due to pain secondary to traumatic thoracic vertebra fracture, and 2 cases were due to post-operative pain for correction of scoliosis.

These results suggest that although the ESPB analgesic effect may differ slightly, overall, this effect is comparable between patients with pain originating from oncological and non-oncological sources and that ESPB can have a potential role in treating pain with these conditions.

A second subgroup analysis, based on the dominant pain type, did not reveal significant differences in either the pre- or post-pain NRS score nor in the portion of patients who reported more than 50% improvement.

When the pre- and post-NRS scores were compared within the mixed pain [14] group, the improvement did not reach statistical significance (*p*-value = 0.334). However, due to the small number of patients in this subgroup, this finding should be interpreted carefully, and conclusions should not be drawn.

An analysis of the chronicity of the pain experience duration was also carried out. More than 60% of the study population experienced pain for more than a year prior to the execution of the ESPB. Given that our clinic serves as a tertiary center, often with long waiting times, such a proportion is expected. Although all three subgroups reported statistically significant NRS reduction, the largest proportion of patients with pain reduction of more than 50% was found in the patients with symptom durations of less than four months. These results support previous findings regarding the importance of early treatment and support the recommendation of early referral to a pain specialist for early intervention [20,21].

### 4.1. Procedure-Related Aspects

As mentioned above, much is yet unknown about thoracic ESPB. Not only regarding its indications and efficacy but also various technical aspects. As the procedure is a single shot, not a continuous infusion, the dosage delivered should not exceed the recommended daily dosage. We use 10 mL of 1% lidocaine with dexamethasone 10 mg for a unilateral injection and 15–20 mL of 1% lidocaine with dexamethasone 10 mg for a bilateral procedure. Corticosteroid has an established role in the management of both neuropathic and cancer pain [22,23,24]. The choice of Dexamethasone is for two sets of reasons. As a corticosteroid, it has high potency, a long duration of action, and minimal mineralocorticoid effect. Moreover, the solution is non-particulate; hence, it confers a lower risk of vascular damage in the thoracic area and is the recommended corticosteroid for thoracic injection [25].

### 4.2. Safety Aspects

Most chronic pain patients are treated at an ambulatory outpatient clinic. This should be considered when evaluating the approach and safety aspects of such a procedure.

In the immediate vicinity of the needle performing the block, there are no neuro-vascular structures at risk. To date, there have been minimal procedure-related complications reported with this block compared to the traditional thoracic neuraxial blocks [8,26,27]. Our data support this notion, as no major adverse effects were recorded. A total of 145 procedures were carried out, of which 45% were in a bilateral manner. The only adverse effects reported were a systemic response to steroids consisting of a mild and expected transient increased level of blood glucose level and increased blood pressure, none of which required further investigation or hospital admission.

The classification of the American Society of Regional Anesthesia (ASRA) for pain procedures considers musculoskeletal injections and thoracic facet medial branch block as procedures with a low risk for bleeding [28,29]. Recent reports also suggest a low risk of bleeding from the ESPB [30]. However, some patients are at higher risk due to various co-morbidities, and hence, we perform a personal stratification of risk for bleeding for each patient before recommending the procedure. Three patients underwent the procedure while treated with Enoxaparin at therapeutic dosage. Those patients were instructed to stay on bed rest for an hour following the procedure and were later discharged without adverse effects.

### 4.3. Post-Procedure Aspects

All patients undergoing lower thoracic ESPB are tested for motor function following the procedure to screen for any unintended motor weakness. However, we do not carry out sensory tests to evaluate the dermatomal coverage.

We do not perform additional procedures before evaluating the response of the first procedure. A follow-up is routinely scheduled 6–8 weeks after the procedure. A patient that reports a substantial improvement is discharged with a set of recommendations for future maintenance by their primary care physician.

### 4.4. Limitations

This study is a single-center trial and is limited by its retrospective nature. Part of the challenge associated with retrospective analyses is the possibility that pharmaceutical changes or other manual manipulations unbeknown to the team might affect the outcomes. However, in our pain institute, we avoid the use of pharmaceutical changes following ESP in order to allow for precise estimation of the analgesic effect of the procedure for future reference in cases of additional pain management advice. Even though a substantial percentage presented an NRS improvement exceeding 50%, our results should be interpreted carefully due to the sample size of the study. Another limitation that future studies should address is the long follow-up time, as the positive effect of the block may wane over time. Furthermore, a distinction of possible spinal anatomical structures and mechanisms (i.e., facet joint degeneration, discogenic changes, etc.) is missing and should be explored on a larger scale study.

In summary, thoracic pain is common and could lead to substantial disability and other negative impacts on the patient’s life and society. It was argued that thoracic pain should be considered a discrete and important clinical entity, independent of pain experienced in other areas of the spine [31]. This study was conducted with the aim of better understanding and providing pain management for thoracic pain.

## 5. Conclusions

Thoracic ESPB is a simple and safe technique. The results support the possible role of ESPB for chronic as well as cancer-related pain.

Due to the simplicity of the ESPB, it could potentially be applied in multiple disciplines and setups, such as the emergency department and at an ambulatory practice.

In the future, prospective trials should be carried out to expand our knowledge and determine the proper and safe application of this type of block.

## Figures and Tables

**Table 1:** Demographic and preprocedural variables.

(A) Etiology				
	Non-Cancer Related	Cancer Related	*p*-Value	Total
**Total**	66	44		110
**Male**	26 (39%)	17 (39%)	0.92	43 (39%)
**Age**	61.8 ± 16.9	60.4 ± 14.8	0.26	61.2 ± 16.1
**Bilateral injection**	29 (44%)	20 (45%)	0.91	49 (45%)
**Num of injections**				
**1**	46 (70%)	26 (59%)		72 (65%)
**2**	18 (27%)	17 (39%)		35 (32%)
**3**	2 (3%)	1 (2%)	0.48	3 (3%)
**Follow-up (weeks)**	8.1 ± 2.8	7.6 ± 2.2	0.5	7.9 ± 4.6
**(B) Dominant Pain Type**				
	**Nociceptive**	**Neuropathic**	**Mixed**	***p*** **-Value**
**Total**	55	45	10	
**Male**	18 (33%)	22 (49%)	3 (30%)	0.223
**Age**	62.4 ± 15.1	59.8 ± 18.3	61.1 ± 9.7	0.710
**Bilateral injection**	32 (58%)	17 (38%)	0 (0%)	0.003
**Num of injections**				
**1**	37 (67%)	26 (58%)	9 (90%)	
**2**	17 (31%)	17 (38%)	1 (10%)	
**3**	1 (2%)	2 (4%)	0 (0%)	0.348
**Follow-up (weeks)**	8.0 ± 4.5	8.1 ± 5.1	6.3 ± 2.6	0.570
**(C) Duration of Pain**				
	**≤4 Months**	**4–12 Months**	**≥12 Months**	***p*** **-Value**
**Total**	20	22	68	
**Male**	6 (30%)	12 (55%)	25 (37%)	0.238
**Age**	68.1 ± 15.5	64.0 ± 12.9	58.3 ± 16.5	0.1
**Bilateral injection**	5 (25%)	5 (23%)	39 (57%)	0.001
**Num of injections**				
**1**	16 (80%)	14 (64%)	42 (62%)	
**2**	4 (20%)	8 (36%)	23 (34%)	
**3**	0 (0%)	0 (0%)	3 (4%)	0.568
**Follow-up (weeks)**	6.5 ± 3.7	7.9 ± 5.5	8.3 ± 4.5	0.37

**Table 2:** Level of injection.

Level	Number of Injections
**Total**	110
**T2**	5 (5%)
**T3**	26 (24%)
**T4**	8 (7%)
**T5**	10 (9%)
**T6**	13 (12%)
**T7**	15 (14%)
**T8**	7 (6%)
**T9**	5 (5%)
**T10**	12 (11%)
**T11**	3 (3%)
**T12**	6 (5%)

**Table 3:** Average pain intensity.

(A) Etiology					
	Non-Cancer Related	Cancer Related		Total	*p*-Value
**Pre NRS**	7.7 ± 1.4	7.1 ± 1.3		7.4 ± 1.4	0.88
**Post NRS**	5.1 ± 2.8	5.0 ± 2.3		5.0 ± 2.6	0.077
***p*-values (pre vs. post** **procedure)**	>0.001	>0.001		>0.001	
**NRS improved >2.5**	37 (56%)	21 (48%)		58 (53%)	0.44
**NRS improved >50%**	32 (48%)	13 (30%)		45 (41%)	0.051
**(B) Dominant Pain Type**					
	**Nociceptive**	**Neuropathic**	**Mixed**	**Total**	***p*-Value**
**Pre NRS**	7.6 ± 1.3	7.4 ± 1.4	6.9 ± 1.6	7.4 ± 1.4	0.71
**Post NRS**	5.0 ± 2.3	5.0 ± 2.5	5.7 ± 4.5	5.0 ± 2.6	0.19
***p*-values (pre vs. post** **procedure)**	>0.001	>0.001	0.334	>0.001	
**NRS improved >2.5**	32 (58%)	22 (49%)	4 (40%)	58 (53%)	0.45
**NRS improved >50%**	24 (44%)	17 (38%)	4 (40%)	45 (41%)	0.92
**(C) Duration of Pain**					
	**≤4 Months**	**4–12 Months**	**≥12 Months**	**Total**	***p*-Value**
**Pre NRS**	7.1 ± 1.7	7.1 ± 1.1	7.6 ± 1.4	7.4 ± 1.4	0.164
**Post NRS**	4.7 ± 3.7	4.4 ± 2.4	5.5 ± 2.3	5.0 ± 2.6	0.276
***p*-values (pre vs. post** **procedure)**	0.01	>0.001	>0.001	>0.001	
**NRS improved >2.5**	12 (60%)	15 (68%)	31 (46%)	58 (53%)	0.14
**NRS improved >50%**	12 (60%)	12 (55%)	21 (31%)	45 (41%)	0.02

## Data Availability

The data presented in this study are available on request from the corresponding author.
